# Relationship between the dynamics of non-alcoholic fatty liver disease and incident diabetes mellitus

**DOI:** 10.1038/s41598-022-06205-8

**Published:** 2022-02-15

**Authors:** Ji Eun Han, Han-Bit Shin, Young Hwan Ahn, Hyo Jung Cho, Jae Youn Cheong, Bumhee Park, Soon Sun Kim

**Affiliations:** 1 Department of Gastroenterology, Ajou University School of Medicine, 164 Worldcup-ro, Yeongtong-gu, Suwon, Gyeonggi-do 16499 Republic of Korea; 2 Office of Biostatistics, Ajou Research Institute for Innovation Medicine, Ajou University Medical Center, Suwon, Republic of Korea; 3 Departments of Biomedical Informatics, Ajou Research Institute for Innovative Medicine, Ajou University School of Medicine, Suwon, Republic of Korea

**Keywords:** Diseases, Endocrinology, Medical research

## Abstract

The aim of the current study was to evaluate the association between changes in non-alcoholic fatty liver disease (NAFLD) over time and risk of incident diabetes mellitus (DM). In total, 3047 subjects without underlying DM were followed up for 14 years from the Anseong-Ansan cohort. NAFLD status was determined biennially using the hepatic steatosis index (HSI), and subjects were clustered into seven groups according to changes in HSI, body mass index (BMI), and homeostatic model assessment of insulin resistance (HOMA-IR): none, persistent, transient, transient resolved, resolved, incident, and recurrent NAFLD (Groups 1–7, respectively). Predictive abilities were compared between the dynamics of HSI and single time points. Regarding the changes in HSI, the risk of incident DM was highest in Group 2 (hazard ratio [HR] 2.710; *P* < 0.001), followed by Groups 7 (HR 2.062; *P* < 0.001) and 3 (HR 1.559; *P* = 0.027). The predictive ability for DM was powerful in order of HOMA-IR, HSI and BMI. The dynamics of NAFLD were less predictive of incident DM than single time-point NAFLD. In conclusion, NAFLD is more useful than BMI in predicting incident DM. However, NAFLD status at single time points can better predict incident DM than dynamic changes in HSI.

## Introduction

Non-alcoholic fatty liver disease (NAFLD) is the most common cause of liver disease worldwide, with an estimated prevalence ranging from 25 to 45%, increasing in parallel with that of obesity and diabetes. NAFLD is a hepatic manifestation of metabolic syndrome and is strongly associated with hepatic insulin resistance. NAFLD, especially non-alcoholic steatohepatitis (NASH), is associated with an increased risk of type II diabetes mellitus (DM) and increased morbidity and mortality related to liver and cardiovascular disease (CVD)^1–7^.

The relationship between NAFLD and type II DM is dynamic and reversible. A study to assess the risk of incident DM with variable liver statuses over time determined that their sustained NAFLD group had a significantly increased risk compared with the never NAFLD and intermittent NAFLD groups^8^. Meanwhile, the risk of incident DM could be decreased by the resolution of NAFLD^9^. However, there has been no study regarding the effect of dynamic change in NAFLD status over time on the risk of incident DM.

The Korea Disease Control and Prevention agency started a large-scale cohort project called the Korean Genome and Epidemiology Study (KoGES) in 2001 to investigate the genetic and environmental factors related to chronic diseases such as DM, hypertension, obesity, metabolic syndrome, and CVD, which are common among Koreans. The Anseong-Ansan cohort, a part of KoGES, is a representative community-based cohort. The subjects of this study were selected from the general population living in Anseong and Ansan cities in Korea, who were followed up for the development of chronic diseases including DM^10^.

NAFLD is a disease that requires long-term follow-up because it can cause extrahepatic complications after a long duration. Therefore, even though it may be simple and easy to evaluate the risk of DM or CVD development based on the state of hepatic steatosis at any time point, it may not reflect the dynamic changes in hepatic steatosis status in detail during long-term follow-up. For these reasons, if patients with NAFLD are classified into well-defined groups and stratified as per the changes in hepatic steatosis status over time, differentiated intervention can be provided for each group in clinical practice.

Therefore, we aimed to classify patients with NAFLD according to the pattern of changes in NAFLD status over time using cluster analysis and to predict incident risk of DM in each group using the Anseong-Ansan cohort datasets.

## Results

### Baseline characteristics of the subjects

**Figure 1:**
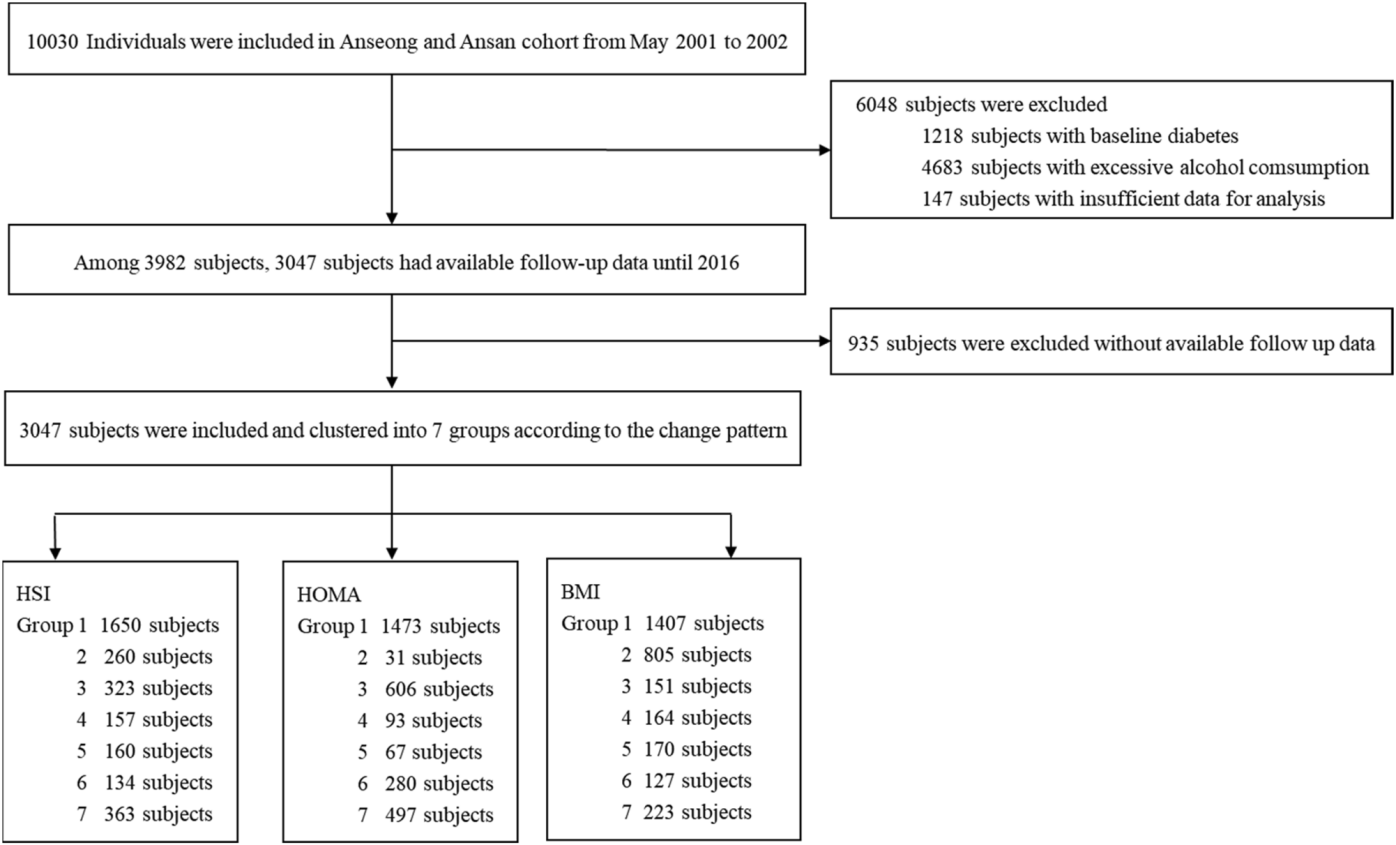
Flow chart of subject inclusion and exclusion. *HSI* hepatic steatosis index, *HOMA-IR* homeostatic model assessment for insulin resistance, *BMI* body mass index.

**Figure 2:**
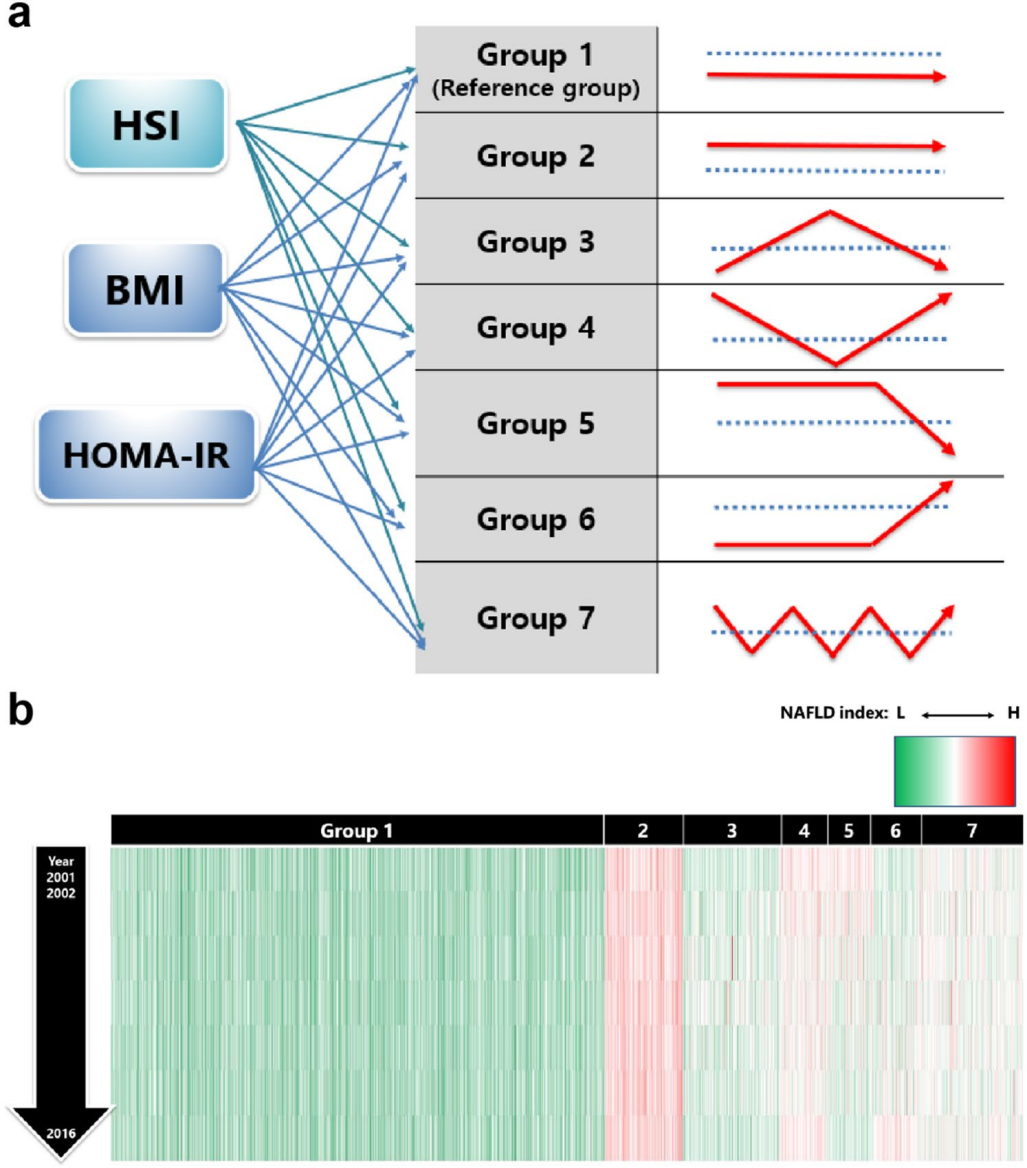
Clustering analysis and heatmap visualization according to HSI clustering. (**a**) Clustering analysis according to change pattern of HSI, BMI, and HOMA-IR. Reference value for NAFLD, obesity, and insulin resistance were defined as an HSI of ≥ 36, BMI of ≥ 25 kg/m^2^, and HOMA-IR of ≥ 2.5, respectively. Subjects was divided into seven groups by clustering. Group 1, subjects who had no NAFLD; Group 2, subjects with persistent NAFLD; Group 3, subjects who had only NAFLD once at the mid-point of the follow-up period; Group 4, subjects who had NAFLD from the beginning and showed resolution only once at the mid-point of the follow-up period; Group 5, subjects who had NAFLD from the beginning but resolved in the last year; Group 6, subjects did not have NAFLD from the beginning but developed it in the last year; and Group 7, subjects with repeated development and improvement of NAFLD. (**b**) Heatmaps for clustering according to change pattern of HSI. The y-axis represents the observed year during the follow-up period and the x-axis represents the group classification. As the value for each year changed from lower to higher value, it is highlighted green to red on the heatmap. *HSI* hepatic steatosis index, *BMI* body mass index, *HOMA-IR* homeostatic model assessment for insulin resistance, *NAFLD* non-alcoholic fatty liver disease.

The baseline characteristics of the subjects are presented in Table 1 and Supplementary Tables S1–S3 online. The subjects comprised 1025 men (33.6%) and 2022 women (66.4%) aged 51.5 ± 8.3 years. The subjects were further divided according to the changes in their NAFLD status over time (Fig. 1a). Females were predominant in all groups. The persistent NAFLD group (Group 2) tended to have higher body mass index (BMI), Waist circumference (WC), fasting plasma glucose levels, hemoglobin A1c (HbA1c) levels, and blood pressure at baseline than the other groups. Group 5 tended to have higher levels of gamma-glutamyl transpeptidase (GGT), aspartate aminotransferase (AST), alanine aminotransferase (ALT), homeostatic model assessment of insulin resistance (HOMA-IR), and triglyceride (TG) at baseline than the other groups. Further, Group 6 showed the highest TG/high-density lipoprotein (HDL) ratio. We visualized hepatic steatosis index (HSI), BMI, and HOMA-IR values of each group using heatmaps (Fig. 2b and Supplementary Fig. S1 online). By visualizing the pattern of change in HSI over time with a heatmap, the characteristics of the change pattern between each group could be well distinguished, and this was the same for BMI and HOMA-IR.

**Table 1:** Baseline characteristics in HSI group.

	All patients (n = 3047)							*P*-value*
	Group 1	Group 2	Group 3	Group 4	Group 5	Group 6	Group 7	
No. of patients	1,650	260	323	157	160	134	363	
Age, year	52.0 ± 8.6	51.2 ± 7.8	50.8 ± 7.8	50.6 ± 7.8	51.9 ± 8.0	49.4 ± 8.0	50.7 ± 7.8	0.0007
Male sex, %	33.76	26.92	33.75	35.03	39.38	21.64	39.12	0.0016
BMI, kg/m^2^	22.8 ± 2.1	29.8 ± 2.3	24.6 ± 1.7	27.4 ± 1.7	26.9 ± 2.0	25.1 ± 1.8	26.2 ± 1.8	< 0.0001
WC, cm	77.8 ± 7.3	93.4 ± 7.9	82.2 ± 6.6	88.4 ± 6.5	87.8 ± 7.8	82.7 ± 7.2	85.8 ± 6.3	< 0.0001
SBP, mmHg	120.4 ± 17.8	128.5 ± 16.9	122.7 ± 16.6	124.2 ± 17.9	127.1 ± 18.0	119.5 ± 17.1	123.7 ± 17.4	< 0.0001
DBP, mmHg	79.7 ± 11	86.0 ± 10.6	81.5 ± 10.6	85.0 ± 11.3	83.6 ± 10.8	80.0 ± 10.6	82.6 ± 10.8	< 0.0001
Glucose, mg/dL	81.1 ± 7.9	83.7 ± 8.5	82.3 ± 8.3	83.7 ± 7.8	83.5 ± 8.6	80.7 ± 8.2	83.3 ± 8.9	< 0.0001
Creatinine, mg/dL	0.8 ± 0.2	0.8 ± 0.7	0.8 ± 0.2	0.8 ± 0.2	0.8 ± 0.2	0.8 ± 0.2	0.8 ± 0.2	< 0.0001
Platelet, × mm^9^	260.3 ± 61.4	276.7 ± 59.1	268.2 ± 59.5	276.4 ± 61.1	277.3 ± 71.8	274.1 ± 61.1	276.2 ± 64.1	< 0.0001
Albumin, g/dL	4.2 ± 0.3	4.2 ± 0.3	4.2 ± 0.3	4.2 ± 0.3	4.2 ± 0.3	4.2 ± 0.3	4.2 ± 0.3	0.8647
GGT, U/L	17.2 ± 18.4	29.9 ± 27.0	19.4 ± 15.5	28.0 ± 26.1	32.5 ± 27.9	21.1 ± 21.4	30.3 ± 103.6	< 0.0001
AST, U/L	26.4 ± 8.5	29.2 ± 10.4	27.7 ± 22.7	30.2 ± 20.5	32.0 ± 17.7	25.1 ± 6.8	27.6 ± 9.0	< 0.0001
ALT, U/L	20.7 ± 10.7	33.3 ± 19.3	24.6 ± 15.5	36.0 ± 30.2	40.2 ± 28.0	20.1 ± 7.5	28.7 ± 17.3	< 0.0001
Total cholesterol, mg/dL	185.7 ± 32.8	195.8 ± 32.4	190 ± 33.1	194.5 ± 35.3	197.1 ± 32.5	193.9 ± 36.8	194.0 ± 32.7	< 0.0001
HDL, mg/dL	46.2 ± 10.3	42.4 ± 8.7	43.7 ± 8.8	40.8 ± 8.3	41.1 ± 9.2	45.4 ± 9.5	42.1 ± 8.5	< 0.0001
TG, mg/dL	132.5 ± 69.2	174.4 ± 97.4	149.3 ± 76.4	180.6 ± 88.7	186.0 ± 93.7	145.4 ± 83.6	167.5 ± 102.8	< 0.0001
TG/HDL	3.7 ± 2.8	3.5 ± 2.2	3.8 ± 2.8	3.4 ± 2.2	3.4 ± 2.2	4.1 ± 2.9	3.6 ± 2.6	0.2221
LDL, mg/dL	115.4 ± 31.0	112.7 ± 27.6	117.6 ± 30.8	120.1 ± 34.0	114.9 ± 32.4	110.5 ± 27.7	114.6 ± 29.5	0.0846
HbA1c, %	5.5 ± 0.3	5.6 ± 0.4	5.5 ± 0.3	5.6 ± 0.4	5.6 ± 0.4	5.5 ± 0.3	5.6 ± 0.3	< 0.0001
HOMA-IR	1.4 ± 1.0	2.0 ± 0.9	1.5 ± 1.1	1.9 ± 1.1	2.0 ± 1.2	1.6 ± 0.8	1.7 ± 0.8	< 0.0001
HSI	30.7 ± 2.6	40.3 ± 3.0	33.2 ± 1.9	38.2 ± 2.0	38.3 ± 2.2	33.6 ± 2.6	35.9 ± 2.5	< 0.0001

Data are presented as mean ± SD.

*P-value using Scheffe as the post hoc analysis for comparing the groups divided by change pattern of NAFLD status over time.

*HSI* hepatic steatosis index, *BMI* body mass index, *WC* waist circumference, *SBP* systolic blood pressure, *DBP* diastolic blood pressure, *GGT* gamma-glutamyl transferase, *AST* aspartate aminotransferase, *ALT* alanine aminotransferase, *HDL* high-density lipoprotein, *TG* triglyceride, *LDL* low-density lipoprotein, *HbA1c* hemoglobin A1c, *HOMA-IR* homeostatic model assessment for insulin resistance, *NAFLD* non-alcoholic fatty liver disease.

### Risk factors for developing incident DM

In univariate analysis, older age, higher WC, higher BMI, higher fasting glucose level, higher HbA1c level, higher HOMA-IR level, lower HDL cholesterol level, higher TG level, higher TG/HDL ratio, underlying hypertension, higher ALT level (≥ 19 U/L for women, ≥ 30 U/L for men), higher AST level, and higher GGT level (> 60 U/L) were identified as considerable risk factors for predicting DM development. Multivariate regression analysis was performed using all variables identified as risk factors for DM in the univariate analysis (model 1) and, additionally, using selected variables sorted out through stepwise selection (model 2). A higher level of fasting blood glucose was a risk factor for incident DM in model 1 in multivariate analysis, which was adjusted for all variables. A higher level of fasting blood glucose and the TG/HDL ratio were risk factors for incident DM in model 2 in multivariate analysis, which was adjusted for selected variables (HbA1c level, blood glucose level, HSI, TG/HDL ratio, GGT, and diastolic blood pressure), including changes in HSI, BMI, and HOMA-IR (Table 2).

**Table 2:** Risk factors for developing type II DM.

	Univariate analysis		Multivariate analysis (model1)		Multivariate analysis (model 2)	
	HR (95% CI)	*P*-value	HR (95% CI)	*P*-value	HR (95% CI)	*P*-value*
Age, year	1.403 (1.095–1.797)	0.0074	1.001 (0.986–1.016)	0.9239		
Male sex	1.00 (0.808–1.238)	1				
BMI, kg/m^2^	2.469 (2.018–3.054)	< 0.0001	0.979 (0.857–1.118)			
WC (> 80 cm for women, > 90 cm for men)	2.364 (1.921–2.908)	< 0.0001	1.009 (0.989–1.029)	0.3648		
SBP, mmHg	1.710 (1.396–2.094)	< 0.0001	1.006 (0.997–1.016)	0.1858		
DBP, mmHg	1.701 (1.391–2.081)	< 0.0001	0.998 (0.983–1.013)	0.7791	1.005 (0.996–1.015)	0.2899
Glucose	5.974 (4.435–8.047)	< 0.0001	1.063 (1.051–1.076)	< 0.0001	1.055 (1.044–1.066)	< 0.0001
GGT, U/L	1.977 (1.318–2.966)	0.001	1.000 (0.999–1.001)	0.6922	1.000 (0.999–1.001)	0.6735
AST, U/L	1.855 (1.310–2.626)	0.0005	0.987 (0.957–1.011)	0.3952		
ALT, U/L	2.119 (1.718–2.613)	< 0.0001	1.013 (0.99–1.037)	0.2616		
Total cholesterol, mg/dL	1.350 (1.102–1.654)	0.0037	0.996 (0.992–1.000)	0.0456		
TG/HDL	2.592 (2.085–3.223)	< 0.0001	0.971 (0.817–1.153)	0.7339	1.041 (1.012–1.072)	0.006
LDL, mg/dL	1.021 (0.821–1.269)	0.8536				
HDL, mg/dL	1.777 (1.418–2.226)	< 0.0001	0.988 (0.970–1.007)	0.2144		
TG, mg/dL	2,543 (2.075–3.116)	< 0.0001	1.002 (0.997–1.007)	0.4484		
HbA1c, %	7.005 (5.501–8.919)	< 0.0001	13.992 (9.72–20.142)	< 0.0001	14.158 (10.029–19.988)	< 0.0001
HOMA-IR	2.736 (2.138–3.501)	< 0.0001	0.887 (0.768–1.024)	0.1004		
HSI	3.053 (2.496–3.735)	< 0.0001	0.973 (0.867–1.092)	0.6457	0.996 (0.954–1.040)	0.8722

*P-value using Scheffe as the post hoc analysis for comparing the groups divided by change pattern of NAFLD status over time. Model 1 was adjusted for age, blood glucose level, ALT, AST, GGT, total cholesterol, HDL, TG, HbA1C, platelet, WC, SBP, DBP, BMI, HOMA-IR, and HSI, including changes in HSI, HOMA-IR, and BMI. Model 2 was adjusted for HbA1c, blood glucose level, HSI, TG/HDL ratio, GGT, and DBP (stepwise selection), including changes in HSI, HOMA-IR, and BMI.

*DM* diabetes mellitus, *HR* hazard ratio, *CI* confidence interval, *BMI* body mass index, *WC* waist circumference, *SBP* systolic blood pressure, *DBP* diastolic blood pressure, *GGT* gamma-glutamyl transferase, *AST* aspartate aminotransferase, *ALT* alanine aminotransferase, *TG* triglyceride, *HDL* high-density lipoprotein, *LDL* low-density lipoprotein, *HbA1c* hemoglobin A1c, *HOMA-IR* homeostatic model assessment for insulin resistance, *HSI* hepatic steatosis index, *NAFLD* non-alcoholic fatty liver disease.

### Comparison of the risk of developing incident DM according to change pattern of HSI and metabolic associated fatty liver disease (MAFLD)

**Figure 3:**
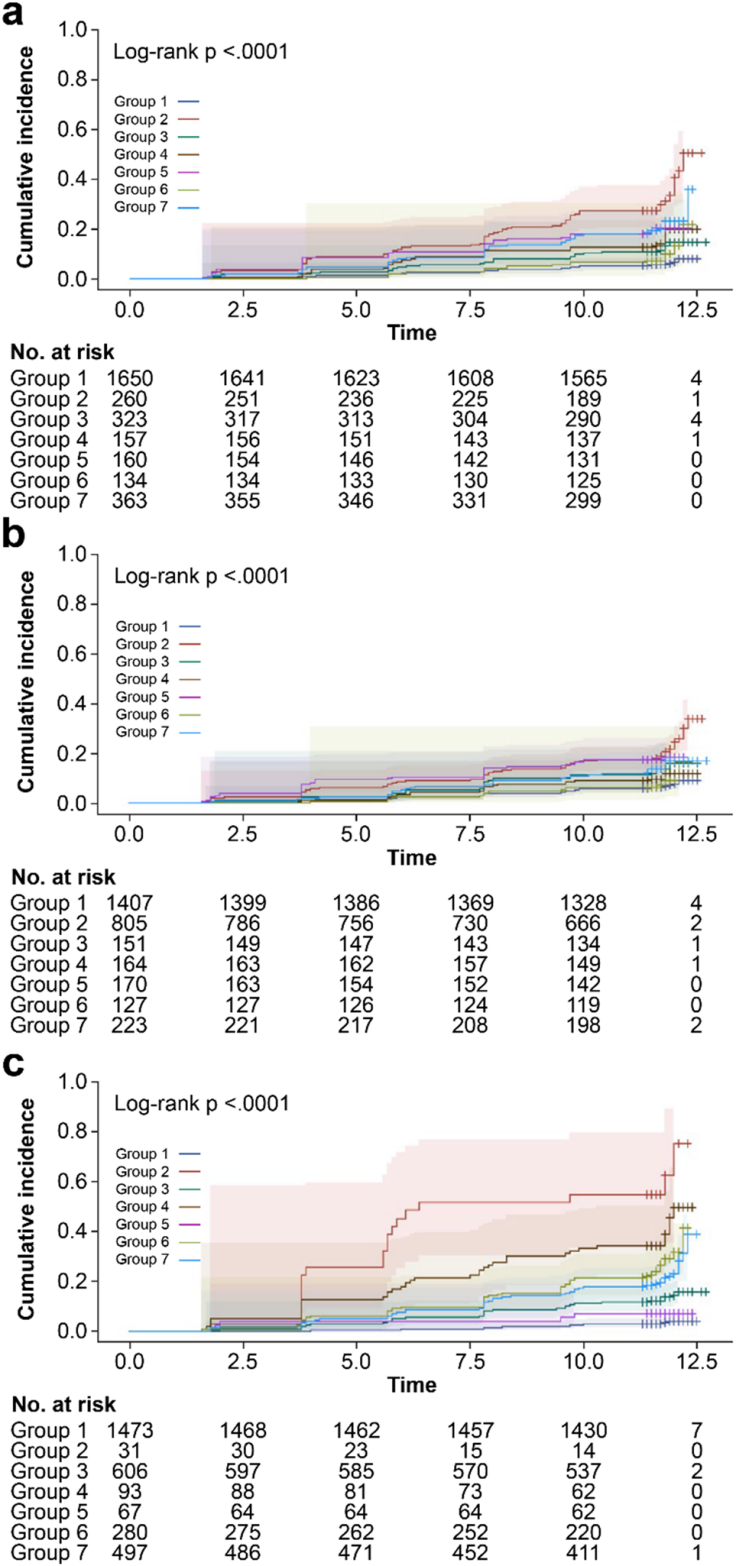
Comparison of diabetes mellitus development by Kaplan–Meier curves in included subjects according to groups divided by change pattern of non-alcoholic fatty liver disease (NAFLD) status as assessed by hepatic steatosis index (HSI), body mass index (BMI) and Homeostatic model assessment for insulin resistance (HOMA-IR). (**a**) Kaplan–Meier curves of diabetes mellitus (DM) development according to NAFLD status change. (**b**) Kaplan–Meier curves of DM development according to BMI status change. (**c**) Kaplan–Meier curves of DM development according to HOMA-IR status change.

We compared the risk of incident DM between the HSI groups. In the multivariate analysis (model 1), the risk of incident DM was substantially higher in the groups in the following order: Group 2, 7, and 3. In model 2, the persistent NAFLD group showed the highest risk of DM. The risk of incident DM was higher in all groups, except Groups 4 and 6, than in the reference group in the following order: Group 2 (persistent NAFLD; hazard ratio [HR] 3.047; 95% confidence interval [CI] 1.755–5.289; *P* < 0.001), Group 7 (recurrent NAFLD; HR 2.259; 95% CI 1.505–3.391; *P* < 0.001), Group 5 (resolved NAFLD; HR 1.863; 95% CI 1.111–3.123; *P* = 0.0183), and Group 3 (transient NAFLD; HR 1.625; 95% CI 1.096–2.41; *P* = 0.0223) (Table 3 and Fig. 3a).

**Table 3:** Comparing incident DM risk between HSI groups.

	Univariate analysis		Multivariate analysis (model 1)		Multivariate analysis (model 2)	
	HR (95% CI)	*P*-value	HR (95% CI)	*P*-value	HR (95% CI)	*P*-value*
Group 1	Ref.					
Group 2	6.118 (4.594–8.148)	< 0.0001	3.059 (1.747–5.355)	< 0.0001	3.047 (1.755–5.289)	< 0.0001
Group 3	2.105 (1.466–3.023)	< 0.0001	1.572 (1.053–2.347)	0.0269	1.625 (1.096–2.410)	0.0223
Group 4	2.909 (1.904–4.445)	< 0.0001	1.613 (0.980–2.852)	0.1003	1.672 (0.958–2.918)	0.0706
Group 5	3.634 (2.444–5.404)	< 0.0001	1.672 (0.980–2.852)	0.0591	1.863 (1.111–3.213)	0.0183
Group 6	1.651 (0.945–2.886)	0.0783	1.423 (0.785–2.579)	0.2454	1.381 (0.765–2.495)	0.2841
Group 7	3.786 (2.820–5.081)	< 0.0001	2.127 (1.404–3.221)	0.0004	2.259 (1.505–3.391)	< 0.0001

Model 1 was adjusted for age, blood glucose level, ALT, AST, GGT, total cholesterol, HDL, TG, HbA1C, platelet, WC, SBP, DBP, BMI, HOMA-IR and HSI, including changes in HSI, HOMA-IR, and BMI. Model 2 was adjusted for HbA1c, blood glucose level, HSI, TG/HDL ratio, GGT, and DBP (stepwise selection), including changes in HSI, HOMA-IR, and BMI.

*DM* diabetes mellitus, *HSI* hepatic steatosis index, *HR* hazard ratio, *CI* confidence interval, *ALT* alanine aminotransferase, *AST* aspartate aminotransferase, *GGT* gamma-glutamyl transferase, *HDL* high-density lipoprotein, *TG* triglyceride, *HbA1c* hemoglobin A1c, *SBP* systolic blood pressure, *DBP* diastolic blood pressure, *BMI* body mass index, *HOMA-IR* homeostatic model assessment of insulin resistance.

In regard to change pattern of MAFLD, multivariate analysis (model 1 and 2) showed that the risk of incident DM was substantially higher in the groups in the following order: Group 2, 7, and 3. The risk of incident DM was highest in Group 2 (HR 2.405; P < 0.001), followed by Group 7 (HR 1.811; P = 0.0002) and 3 (HR 1.46; P = 0.0185) in model 2 (Supplementary Table S4 online).

### Comparison of the risk of developing incident DM according to change pattern of BMI and HOMA-IR, Fibrosis-4 (FIB-4) score

In the multivariate analysis between BMI groups, none of the groups showed substantially higher risk of DM in model 1, and only Groups 2 and 4 affect the risk of DM compared with the reference group in model 2; Group 2 (HR 0.656; 95% CI 0.439–0.980; *P* = 0.0394) and Group 4 (HR 0.561; 95% CI 0.320–0.984; *P* = 0.0439) (Supplementary Table S5 online and Fig. 3b).

In the multivariate analysis between HOMA-IR groups (all in model 1 and 2), all groups, except Group 5, showed a significantly higher risk of DM than the reference group in the order of Group 2 (persistent insulin resistance [IR] group), Group 6 (incident IR), Group 4 (transient resolved IR), Group 7 (recurrent IR) and Group 3 (transient IR) [Group 2 (HR 8.892; 95% CI 5.067–15.603; *P* < 0.001), Group 6 (HR 5.146; 95% CI 3.524–7.515; *P* < 0.001), Group 4 (HR 3.782; 95% CI 2.373–6.028; *P* < 0.001), Group 7 (HR 3.15; 95% CI 2.21–4.49; *P* < 0.001), Group 3 (HR 2.471; 95% CI 1.728–3.535; *P* < 0.001), and Group 5 (HR 1.147; 95% CI 0.453–2.904; *P* = 0.8036)] (Table 4 and Fig. 3c).

**Table 4:** Comparing incident DM risk between HOMA-IR groups.

	Univariate analysis		Multivariate analysis (model 1)		Multivariate analysis (model 2)	
	HR (95% CI)	*P*-value	HR (95% CI)	*P*-value	HR (95% CI)	*P*-value
Group 1	Ref.					
Group 2	28.157 (16.655–47.601)	< 0.0001	10.328 (5.440–19.609)	< 0.0001	8.892 (5.067–15.603)	< 0.0001
Group 3	3.971 (2.811–5.609)	< 0.0001	2.435 (1.697–3.493)	< 0.0001	2.471 (1.728–3.535)	< 0.0001
Group 4	13.970 (9.178–21.264)	< 0.0001	4.277 (2.493–7.338)	< 0.0001	3.782 (2.373–6.028)	< 0.0001
Group 5	2.097 (0.838–5.247)	0.1134	1.181 (0.450–3.099)	0.7348	1.147 (0.453–2.904)	0.8036
Group 6	8.734 (6.148–12.407)	< 0.0001	5.084 (3.462–7.466)	< 0.0001	5.146 (3.524–7.515)	< 0.0001
Group 7	6.656 (4.794–9.243)	< 0.0001	3.143 (2.174–4.545)	< 0.0001	3.150 (2.210–4.490)	< 0.0001

Model 1 was adjusted for age, blood glucose level, ALT, AST, GGT, total cholesterol, HDL, TG, HbA1C, platelet, WC, SBP, DBP, BMI, HOMA-IR and HSI, including changes in HSI, HOMA-IR, and BMI. Model 2 was adjusted for HbA1c, blood glucose level, HSI, TG/HDL ratio, GGT, and DBP (stepwise selection), including changes in HSI, HOMA-IR, and BMI.

*DM* diabetes mellitus, *HOMA-IR* homeostatic model assessment for insulin resistance, *HR* hazard ratio, *CI* confidence interval, *ALT* alanine aminotransferase, *AST* aspartate aminotransferase, *GGT* gamma-glutamyl transferase, *HDL* high-density lipoprotein, *TG* triglyceride, *HbA1c* hemoglobin A1c, *SBP* systolic blood pressure, *DBP* diastolic blood pressure, *BMI* body mass index, *HSI* hepatic steatosis index.

In the multivariate analysis between FIB-4 groups (all in model 1 and 2), none of the groups showed substantially higher risk of DM than the reference group (Supplementary Table S6 online).

### Comparison of discriminatory ability for predicting incident DM among the change patterns of HSI, BMI and HOMA-IR over time

**Figure 4:**
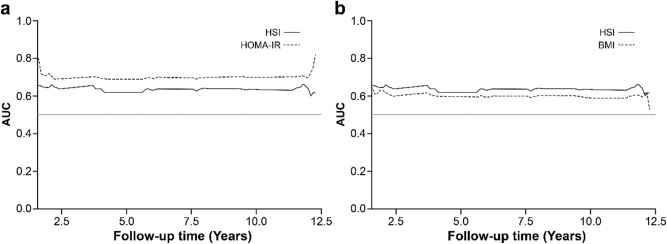
Comparison of integrated AUC among change patterns of HSI, BMI, and HOMA-IR in predicting incident diabetes mellitus (DM). (**a**) Integrated area under the receiver operating characteristic curve (IAUC) plot based on change patterns of HOMA-IR and HSI in predicting incident DM. (**b**) IAUC plot based on change patterns of HSI and BMI in predicting incident DM. *HSI* hepatic steatosis index, *HOMA-IR* homeostatic model assessment for insulin resistance, *BMI* body mass index, *AUC* area under the receiver operating characteristic curve.

To predict which of the three grouping patterns of HSI, BMI, and HOMA-IR was better for predicting the onset of incident DM, we compared the time-dependent area under the curve and Harrell’s C-index for each group. Among the change patterns of HOMA-IR, HSI, and BMI, the predictive ability for incident DM was higher with HOMA-IR than with HSI and BMI [Harrell’s C-index, 0.6942 (*P* < 0.001) vs. 0.6333 (*P* < 0.001) vs. 0.5913 (*P* < 0.001)] (Fig. 4). The differences in Uno’s concordance statistic between HOMA-IR–BMI, HOMA-IR–HSI, and BMI–HSI were 0.1222 (*P* < 0.001), 0.0768 (*P* < 0.001), and − 0.0454 (*P* = 0.0089), respectively.

### Comparison of discriminatory ability for predicting incident DM among the change patterns of HSI and MAFLD over time in MAFLD population

To predict which of the change patterns of HSI and MAFLD was better for predicting the onset of incident DM, we compared the time-dependent area under the curve and Harrell’s C-index for each group. The predictive ability for incident DM was higher with MAFLD than HSI [Harrell’s C-index, 0.6171 (*P* < 0.001) vs. 0.6167 (*P* < 0.001)]. The differences in Uno’s concordance statistic between HSI and MAFLD were − 0.0017 (*P* = 0.1133).

### Comparison of discriminatory ability for predicting incident DM between change pattern of HSI over time and NAFLD development at a single time point

The group that had NAFLD at the first year of the follow-up period had higher predictive ability of incident DM (Harrell’s C = 0.7188, *P* < 0.001) than the group that had NAFLD in the last year of the follow-up period (Harrell’s C = 0.7045, *P* < 0.001). However, there was no considerable difference in the power to explain risk of incident DM, and the difference in Uno’s concordance statistic between the two groups was 0.012 (*P* < 0.9327).

We also compared the predictive ability for incident DM between the change pattern of HSI over time and NAFLD status at a single time point. NAFLD status at both the first and last years of follow-up period could predict incident DM better than the dynamic change in HSI, and the differences in Uno’s concordance statistic between the dynamic change in HSI and NAFLD status at the first and last years of the follow-up period were − 0.0824 (*P* = 0.0001) and − 0.0836 (*P* < 0.001), respectively (Supplementary Fig. S2 online).

## Discussion

Through a long-term and large-scale retrospective study, we predicted the risk of incident DM based on a pattern of changes in NAFLD status over time. We demonstrated that even a single onset of NAFLD during the follow-up period could be an independent risk factor for DM development regardless of the resolution of NAFLD. In addition, recurrent NAFLD increases the risk of incident DM to a similar extent as that of persistent NAFLD. Moreover, we revealed that a pattern of changes in NAFLD status could explain the development of incident DM better than that in BMI. The risk of incident DM could be more precisely predicted with NAFLD status assessment by adding AST and ALT to BMI.

The pathogenesis of NAFLD involves a complex interaction among environmental factors, including diet and exercise, obesity, alteration in microbiota, and predisposing genetic traits. Among these factors, hepatic IR is a key pathophysiological mechanism of NAFLD. Free fatty acids (FFAs) overflow to the liver either from dietary intake or visceral adipose tissue, and impaired hepatic elimination of FFAs leads to excess accumulation of TGs and diacylglycerols (DAGs) in the liver. Excess FFAs might interfere with insulin signaling by causing lysosomal instability with leakage of cathepsin B and induction of the nuclear factor-κB–tumor necrosis factor-α (TNF-α) pathway or activation of the caspase-1-interleukin (IL)-1β/IL-18 pathway through the NALP3 inflammasome. Elevated hepatic DAG levels also promote IR by activating protein kinase C and c-Jun N-terminal kinase. Simultaneously, hepatocytes increase the rate of mitochondrial β-oxidation to limit FFA, and the lipid overload further impairs mitochondrial antioxidant capacity, causing oxidative stress by producing reactive oxygen species and mitochondrial leakage and finally these aggravate insulin resistance^11–16^. Inflammatory cytokines such as TNF-α, IL-6, and adiponectin from adipose tissue are also associated with hepatic IR by various pathways^17–19^.

A strong association between NAFLD and type II DM has been demonstrated in many large-scale epidemiological studies. Many cohort studies have shown that elevated liver enzyme levels can increase the risk of type II DM and that NAFLD diagnosed by ultrasonography is associated with a 33% to five-fold increased risk of type II DM in different populations with various follow-up periods and severity of NAFLD^20–22^. Another study showed that liver fat content of > 10% by a quantitative ultrasound method was associated with increased systemic IR and risk of diabetes^23^. Type II DM is also one of the strongest clinical predictors of the progression of NAFLD to NASH and cirrhosis^24^. The presence of type II DM increases the risk of NASH (two- to three-fold) and advanced liver fibrosis^25^. Furthermore, many cohort studies suggest that the presence of DM alone can increase the risk of developing hepatocellular carcinoma by two- to three-folds^26,27^.

In the current study, the risk of incident DM was the highest in the persistent NAFLD group (Group 2), followed by the recurrent NAFLD group (Group 7), the resolved NAFLD group in which NAFLD persisted and resolved at the end (Group 5), and the incident NAFLD group (Group 3) that had only one episode of NAFLD at the mid-point of the follow-up period. HR of the recurrent NAFLD group was comparable to that of the persistent NAFLD group. This result implies that subjects with repetitive deterioration and improvement of NAFLD are also at risk of developing DM, similar to those with persistent NAFLD. One cohort study of Japanese men also reported that transient remission of NAFLD substantially increased the risk of developing type II DM compared with the complete regression of NAFLD and non-NAFLD groups^28^.

Meanwhile, we showed that subjects with incident NAFLD at the mid-point of follow-up period or those with resolved NAFLD also had an increased risk of incident DM. Even if hepatic steatosis status measured by HSI is normalized, various factors including insulin resistance, visceral obesity, dietary composition such as high-fructose and carbohydrate diet, and insufficient physical activity might remain unresolved and persistently affect the onset of DM through hepatokines and oxidative stress. Laboratory markers including GTP, AST, ALT, TG, HbA1c, and HOMA-IR tended to be the highest in the resolved NAFLD group (Group 5) among all groups at baseline. This group had a lower DM risk than the persistent NAFLD group (Group 2). This might be a consequence of lifestyle intervention based on the results of their serum markers^29,30^. The patients with NAFLD have heterogenous characteristics and the close relationship with metabolic dysfunction such as overnutrition, sedentary lifestyle, hypertension, dyslipidemia, and obesity^31,32^. Recent large-scale studies showed that an alcohol dose of a level of 30 g/day for men and 20 g/day for women, which corresponds to the definition of NAFLD, can also aggravate underlying liver disease. Also, there was no safe dose limit of alcohol consumption about liver disease and liver-related mortality^33^.

Considering this aspects, MAFLD including alcohol intake regardless of the dose of alcohol consumption and metabolic derangement, a new concept of NAFLD was introduced by hepatologist’s consensus in 2020^34,35^.

The patients with MAFLD tended to have more severe metabolic derangements at baseline and poorer clinical outcomes such as incident general obesity, central obesity, DM and cerebrovascular events compared to patients with NAFLD in several retrospective studies^36–38^. Therefore, we tried to analyze the subjects according to change pattern of MAFLD additionally. The risk of incident DM was the highest in the persistent MAFLD group (Group 2), followed by the recurrent MAFLD group (Group 7) and the incident MAFLD group (Group 3) that had only one episode of MAFLD at the mid-point of the follow-up period. These results were similar to NAFLD grouping. Furthermore, there was no significant differences in predictive ability for incident DM.

FIB-4 index is being mainly used as one of the noninvasive markers for hepatic fibrosis in NAFLD patients owing to high negative predictive value. Several studies have demonstrated that the FIB-4 index has limitations in diagnosing advanced fibrosis as value above 2.67 especially subjects without type II DM and discriminating advanced fibrosis in lean, morbidly obese patients with MAFLD and older patients over 65 years of age with NAFLD. However, in most cases except for the above, FIB-4 index has been verified clinically useful for ruling out advanced fibrosis^39–43^. To obtain clearer relationship with the onset of incident DM than hepatic steatosis, we additionally analyzed the dynamics of FIB-4 score and incident DM by dividing into 7 groups in the same way as the HSI. The results showed no significant difference in the risk of incident DM in all 7 groups compared with the reference group. Considering the individual 7-year average FIB-4 score, 1731 out of total 3047 (56.8%) were at low risk of advanced liver fibrosis. Also, there was no difference in prevalence of incident DM between two groups (low risk vs indeterminate and high risk group: 11.9%, 12.9%, p-value = 0.4036). Our cohort study included the local general population under 65 years of age and the degree of hepatic fibrosis was not validated by fibroscan or ultrasonography. We believe that these factors contributed to the non-correlation between the onset of incident DM and advanced liver fibrosis.

Considering the change in HOMA-IR, all groups, except Group 5, showed higher risk of DM than the reference group. Considering that HOMA-IR is an indicator that measures insulin resistance, it is not surprising that Harrell’s C-index was the best for predicting power for DM development among HSI, BMI, and HOMA-IR.

We revealed that a pattern of changes in NAFLD status could explain the development of incident DM better than BMI. Considering changes in BMI, Groups 2 and 4 showed substantially lower risk of DM than the reference group. According to a Korean study, even when the BMI is < 25 kg/m^2^, NAFLD is not uncommon and metabolic abnormalities such as IR and hyperlipidemia are more frequently observed compared with in individuals with normal weight and without non-alcoholic fatty liver in Asia^44,45^. In another Korean study analyzing 5,878 non-obese (BMI < 25 kg/m^2^) and non-diabetic subjects, non-obese NAFLD was present in 27%. When a BMI cutoff value of < 23 kg/m^2^ was used, the prevalence of lean NAFLD remained high at 16%^45^. Considering that the change in weight gain may be involved in NAFLD development through IR even within the normal weight range^46^, it could be more appropriate to lower the BMI cutoff level for obesity to < 25 kg/m^2^.

Contrary to what we expected from this study, dynamic changes in NAFLD status over time did not predict incident DM better than NAFLD status at specific time points. Therefore, a model that can discriminate risk of incident DM between stratified groups needs to be studied further.

This study has several limitations. First, to diagnose NAFLD, ultrasonography or liver biopsy was not performed in this study. In one cohort study, HSI showed comparable diagnostic power for NAFLD with the fatty liver index and NAFLD liver fat score (0.80 for HSI, 0.81 for fatty liver index, and 0.83 for NAFLD liver fat)^47^. Other studies also confirmed the accuracy of HSI for biopsy-proven hepatic steatosis and showed usefulness of HSI as one of noninvasive diagnostic method for NAFLD^48–56^. Second, chronic diseases such as thyroid disease or steatogenic medications such as estrogen and steroids that could cause fatty liver were not considered in the present study. Third, a history of hepatitis B and C infections was not available in the current study. Since data on hepatitis virus infections were considered as personal sensitive information, we could not obtain the data from the Anseong-Ansan cohort databases. Lastly, although smoking history, physical activity level, and dietary intake may influence the development of type II DM, these factors were not analyzed.

## Conclusion

HSI, BMI, and HOMA-IR values changed dynamically during the 14-year follow-up period. NAFLD is more useful in predicting incident DM onset than BMI, even when considering changes over time. Our research findings suggest that even if the fatty liver is resolved or develops transiently, the risk of incident DM could increase. When fatty liver disease was diagnosed according to the MAFLD criteria, the results were similar. Therefore, it is necessary to screen for type II DM for several years even after NAFLD or MAFLD is resolved. In the group in which the development and resolution of NAFLD or MAFLD occur repeatedly, the cause of recurrent NAFLD or MAFLD should be appropriately identified and therapeutic intervention, including lifestyle modification, should be actively engaged to prevent the development of type II DM.

## Methods

### Study population

This was a retrospective cohort study. The design and procedure of the present study were approved by the Institutional Review Board of the Ajou University Hospital, Suwon, South Korea (Approval No. AJIRB-MED-EXP-18-238). The requirement for informed consent was waived by the Institutional Review Board of the Ajou University Hospital, Suwon, South Korea. All methods were performed in accordance with the relevant guidelines and regulations.

A total of 10,030 subjects who were part of the Anseong-Ansan cohort and had medical checkups from 2001 to 2016 were examined. Those who had insufficient data collection or had excessive drinking history (threshold of 20 g/day for women and 30 g/day for men) and those diagnosed with underlying DM were excluded from this study. Only subjects who were followed up regularly biennially for 14 years were included. Finally, 3047 subjects without baseline DM were included and analyzed (Fig. 1).

### Definition of terms

The diagnosis of NAFLD was based on the HSI and assessed seven times during 14 years of follow-up period. Modified HSI was used in this study: modified HSI = 8 × ALT/AST ratio + BMI (+ 2 if female). Modified HSI of ≥ 36 was used to diagnose NAFLD^57–60^. Subjects were clustered into seven groups as per the pattern of change in HSI over time as follows: Group 1 did not develop NAFLD during the follow-up period (non-NAFLD); Group 2 had persistent NAFLD (persistent NAFLD); Group 3 developed NAFLD only once at the mid-point of the follow-up period (transient NAFLD); Group 4 had NAFLD from the beginning and showed resolution only once at the mid-point of the follow-up period (transient resolved NAFLD), Group 5 had NAFLD from the beginning but resolved in the last year (resolved NAFLD); Group 6 did not develop NAFLD at the beginning but developed it in the last year (incident NAFLD); and Group 7 developed repeated but not persistent NAFLD from the beginning to the end (recurrent NAFLD). The patterns of change in BMI and HOMA-IR were clustered and analyzed in the same way as the comparison groups. Additionally, to verify the association between dynamic change of liver fibrosis status over time and incident DM risk, we defined FIB-4 score of 1.3 or higher as indeterminate or high-risk of developing advanced liver fibrosis and analyzed in the same way as above^58,59^.

The enrolled subjects were checked for DM development. Diagnosis of DM was defined as fasting plasma glucose levels of ≥ 126 mg/dL or HbA1c levels of ≥ 6.5%, or when a self-report questionnaire indicated antidiabetic medications. Hypertension was diagnosed using the following cutoff values of blood pressure, ≥ 130 or ≥ 85 mmHg, or when the self-report questionnaire indicated antihypertensive medication use^60–62^.

The definition of MAFLD reflects both metabolic dysfunction and alcohol use and the proposed criteria for a positive diagnosis of MAFLD are based on histological (biopsy), imaging or blood biomarker evidence of fat accumulation in the liver (hepatic steatosis) in addition to one of the following three criteria, namely overweight/obesity, presence of type 2 DM, or evidence of metabolic dysregulation. Since this present study was to investigate the relationship between fatty liver status and the onset of DM, we excluded patients with underlying type 2 DM at baseline. The metabolic dysregulation was defined as the presence of two or more of the following conditions: (a) WC ≥ 90 cm in men and 80 cm in women in Asia (b) Blood pressure ≥ 130/85 mmHg or specific drug treatment. (c) TG ≥ 150 g/dL or specific drug treatment. (d) HDL-C < 40 g/dL for male and < 50 g/dL for female. (e) Prediabetes; fasting glucose levels 100 to 125 mg/dL, or 2-h post-load glucose levels 140 to 199 mg/dL or HbA1c 5.7% to 6.4%. (f) HOMA-IR score ≥ 2.5. (g) C-reactive protein level > 2 mg/L^34^.

### Anthropometric and laboratory evaluation

BMI was calculated as body weight (in kg) divided by the square of height (in meter), expressed in kg/m^2^. WC was measured midway between the lower rib margin and iliac crest with a measuring tape. The cutoff values for high WC were 80 cm for women and 90 cm for men according to the definition of central obesity in the Asia–Pacific area. The baseline and follow-up values of serum AST, ALT, fasting plasma glucose, HbA1c, total cholesterol, TG, HDL cholesterol, LDL cholesterol, GGT, albumin, and platelets were collected from each subject. All laboratory marker levels were measured using a conventional automated analyzer. We used the TG/HDL ratio as a variable instead of a single lipid level as it is known as a surrogate marker of NAFLD. As concentrated and integrative information of the multiple lipid variables reflecting the interaction of single lipid values, the TG/HDL ratio has been found to have a stronger relationship with IR or metabolic syndrome than LDL, total cholesterol, TG, or HDL. Particularly in non-obese NAFLD patients with normal lipid levels among Asians, including Taiwan, China, and Korea, the TG/HDL ratio showed a closer relationship with NAFLD and CVD than single lipid levels^63–66^.

### Statistical analysis

Statistical analysis was performed using SAS version 9.4 (https://www.sas.com/; SAS Institute Inc., Cary, NC, USA) and R software package, R version 3.2.5 (https://www.R-project.org/; R Foundation for Statistical Computing, Vienna, Austria). Continuous variables with normal distribution are expressed as mean ± standard deviation. Kaplan–Meier analysis was performed to compare the risk of incident DM development between the groups. Univariate and multivariate Cox regression analyses were performed to identify the risk factors associated with DM. The results are presented as HR with 95% CI.

Harrell’s C-index was used to assess the discriminatory power of the Cox models. For these exploratory analyses, a *P*-value of < 0.05 was considered statistically significant.

## Supplementary Information

Supplementary Information.

## Data Availability

The Data datasets analyzed in current study are publicly available from the Korean Genome and Epidemiology Study (KoGES; 4851-302), National Research Institute of Health, Centers for Disease Control and Prevention, Ministry for Health and Welfare, Republic of Korea. However, limited data may be disclosed.
