# Supplementary material for: Relationship between the dynamics of non-alcoholic fatty liver disease and incident diabetes mellitus
Source: Sci Rep. 2022 Feb 15;12:2538. doi: 10.1038/s41598-022-06205-8 (PMC8847663; doi:10.1038/s41598-022-06205-8)
Supplement: Supplementary file 1 — Supplementary Information. [file 41598_2022_6205_MOESM1_ESM.docx]

**Supplementary Information**

**Relationship between the dynamics of non-alcoholic fatty liver disease and incident diabetes mellitus**

Ji Eun Han, Han-Bit Shin, Young Hwan Ahn, Hyo Jung Cho, Jae Youn Cheong, Bumhee Park & Soon Sun Kim

**Supplementary Table S1.** Baseline characteristics in BMI group

|  | All patients (n=3,047) | | | | | | | *P*-value^*^ |
| --- | --- | --- | --- | --- | --- | --- | --- | --- |
|  | **Group 1** | **Group 2** | **Group 3** | **Group 4** | **Group 5** | **Group 6** | **Group 7** |  |
| No. of patients | 1407 | 805 | 151 | 164 | 170 | 127 | 223 |  |
| Age, yr | 51.2±8.5 | 51.9±8.0 | 51.3±7.5 | 51.0±7.9 | 53.3±8.4 | 50.5±8.2 | 51.2±8.3 | 0.0376 |
| Male sex (%) | 36.96 | 30.81 | 34.44 | 25.61 | 30.59 | 35.43 | 29.6 | 0.0088 |
| BMI, kg/m^2^ | 22.1±1.7 | 28.1±2.1 | 23.8±1.0 | 26.4±1.1 | 26.1±1.1 | 24.0±0.8 | 25.1±1.1 | <0.0001 |
| Waist circumference (>80 cm for women, >90 cm for men) | 76.6±6.8 | 89.6±7.4 | 80.6±6.2 | 85.1±6.3 | 85.6±6.2 | 81.0±6.8 | 83.3±6.2 | <0.0001 |
| SBP, mmHg | 119.4±17.2 | 126.2±17.6 | 122.0±17.3 | 124.3±18.1 | 127.3±19.0 | 118.1±16.6 | 123.0±17.5 | <0.0001 |
| DBP, mmHg | 79.3±10.6 | 84.4±11.3 | 80.5±11.3 | 82.6±10.8 | 84.0±11.5 | 78.8±10.2 | 81.5±10.3 | <0.0001 |
| Glucose, mg/dL | 81.2±7.8 | 82.3±8.2 | 81.9±9.3 | 82.3±8.3 | 84.5±9.2 | 80.1±7.7 | 82.1±8.6 | <0.0001 |
| Creatinine, mg/dl | 0.8±0.2 | 0.8±0.2 | 0.8±0.2 | 0.8±0.2 | 0.8±0.2 | 0.8±0.2 | 0.2±0.2 | 0.3029 |
| Platelet, × mm^9^ | 259.8±61.9 | 275.6±59.8 | 269.7±65 | 270.6±69.7 | 274.6±59.3 | 261.5±62.2 | 270.3±63.5 | <0.0001 |
| Albumin, g/dL | 4.2±0.3 | 4.2±0.3 | 4.2±0.3 | 4.2±0.3 | 4.2±0.3 | 4.2±0.3 | 4.2±0.3 | 0.3029 |
| AST, U/L | 26.8±13.7 | 28.5±13.1 | 26.3±7.1 | 26.6±6.9 | 28.0±10.3 | 26.6±9 | 27.9±10 | 0.0387 |
| ALT, U/L | 22.6±19.2 | 28.9±20.5 | 22.7±10.7 | 25.2±11.2 | 27.2±16.0 | 23.8±13.6 | 25.9±18.5 | <0.0001 |
| GGT, U/L | 18.3±20 | 24.8±22.8 | 33.7±159.3 | 20.8±15.2 | 24±20.7 | 21.2±22 | 22.3±20.7 | <0.0001 |
| Total cholesterol, mg/dL | 185.5±32.7 | 193.3±33.2 | 189.0±33.4 | 191.1±31.2 | 196.0±33.8 | 190.5±38.0 | 190.0 ±33.1 | <0.0001 |
| HDL, mg/dL | 46.1±10.2 | 42.4±8.6 | 45.2±9.8 | 43.6±8.5 | 42.7±9.9 | 44.9±10.0 | 43.5±10.2 | <0.0001 |
| TG, mg/dL | 131.7±70.2 | 166.1±90.7 | 157.8±97.2 | 153.5±82.2 | 174.7±106.4 | 147.5±70.9 | 152.7±78.3 | <0.0001 |
| TG/HDL | 3.7±2.9 | 3.6±2.5 | 3.3±2.2 | 3.5±2.2 | 4.0±2.8 | 3.4±2.2 | 3.8±2.9 | 0.3155 |
| LDL, mg/dL | 115.5±31.3 | 115.2±29.4 | 120.8±33.1 | 113.5±31.4 | 112.2±29.7 | 115.1±31.0 | 114.7±28.9 | 0.2915 |
| HbA1c | 5.5±0.3 | 5.6±0.3 | 5.5±0.3 | 5.6±0.4 | 5.6±0.3 | 5.5±0.3 | 5.6±0.3 | <0.0001 |
| HOMA-IR | 1.4±0.9 | 1.9±1.3 | 1.4±0.7 | 1.7±0.8 | 1.9±1.3 | 1.3±0.9 | 1.5±0.8 | <0.0001 |
| HSI | 30.4±2.8 | 37.6±3.2 | 32.4±2.2 | 35.6±2.4 | 35.4±2.7 | 32.6±2.1 | 34.0±2.5 | <0.0001 |

Data are presented as number (%) or mean±SD.

^*^*P*-value using Scheffe as the post hoc analysis for comparing the groups divided by change pattern of BMI status over time.

*BMI* body mass index, *SBP* systolic blood pressure, *DBP* diastolic blood pressure, *GGT* gamma-glutamyl transferase, *AST* aspartate aminotransferase, *ALT* alanine aminotransferase, *HDL* high-density lipoprotein, *TG* triglyceride, *LDL* low-density lipoprotein, *HbA1c* hemoglobin A1c, *HOMA-IR* homeostatic model assessment for insulin resistance, *HSI* hepatic steatosis index.

**Supplementary Table S2.** Baseline characteristics in HOMA-IR group

|  | All patients (n=3,047) | | | | | | | *P*-value^*^ |
| --- | --- | --- | --- | --- | --- | --- | --- | --- |
|  | **Group 1** | **Group 2** | **Group 3** | **Group 4** | **Group 5** | **Group 6** | **Group 7** |  |
| No. of patients | 1,473 | 31 | 606 | 93 | 67 | 280 | 497 |  |
| Age, yr | 51.2±8.4 | 51.3±8.2 | 51.2±8.1 | 51.6±9.1 | 52.9±8.4 | 52.4±8.4 | 51.9±8.1 | 0.1888 |
| Male sex (%) | 35.03 | 35.48 | 36.30 | 21.51 | 31.34 | 32.5 | 29.38 | 0.0325 |
| BMI, kg/m^2^ | 23.4±2.7 | 27.4±2.9 | 24.8±2.7 | 27.5±3.4 | 25.3±3.1 | 25.8±2.9 | 26.0±2.8 | <0.0001 |
| Waist circumference (>80 cm for women, >90 cm for men) | 78.7±7.9 | 89.4±8.7 | 82.7±8.3 | 89.4±8.4 | 84.6±8.6 | 85.6±7.8 | 85.9±8.4 | <0.0001 |
| SBP, mmHg | 119.2±16.9 | 128.2±16.9 | 122.9±17.8 | 128.8±20.1 | 125.3±19.2 | 125.3±17.6 | 126.6±17.9 | <0.0001 |
| DBP, mmHg | 79.3±10.6 | 84.0±9.2 | 82.0±11.3 | 85.3±11.8 | 83.0±10.7 | 83.3±11.0 | 83.8±11.1 | <0.0001 |
| Glucose, mg/dL | 80.4±7.4 | 88.2±9.3 | 82.3±8.4 | 88.9±10.4 | 85.6±8.0 | 82.0±7.4 | 84.0±8.7 | <0.0001 |
| Creatinine, mg/dl | 0.8±0.2 | 0.8±0.2 | 0.8±0.2 | 0.8±0.2 | 0.8±0.2 | 0.8±0.2 | 0.8±0.2 | 0.8552 |
| Platelet, × mm^9^ | 260.6±61.4 | 265.7±53.3 | 268.4±61.1 | 282.9±62.7 | 255.7±69.1 | 270.6±58.0 | 279.3±65.4 | <0.0001 |
| Albumin, g/dL | 4.2±0.3 | 4.2±0.3 | 4.2±0.3 | 4.2±0.3 | 4.1±0.3 | 4.2±0.3 | 4.3±0.3 | 0.0036 |
| AST, U/L | 26.8±10.9 | 34.1±13.5 | 28.1±17.5 | 26.6±6.8 | 24.9±8.0 | 28.5±13.5 | 27.5±9.3 | 0.0028 |
| ALT, U/L | 22.5±15.7 | 40.1±23.8 | 25.9±23.8 | 29.5±15.8 | 23.9±16.0 | 28.9±21.4 | 27.5±17.1 | <0.0001 |
| GGT, U/L | 17.9±20.1 | 36.4±30.5 | 24.8±80.9 | 25.2±18.6 | 19.6±14.5 | 26.7±24.3 | 24.8±23.3 | <0.0001 |
| Total cholesterol, mg/dL | 186.3±32.8 | 202.8±40.4 | 191.6±33.9 | 194.0±33.5 | 187.6±35.1 | 191.6±32.7 | 193.5±32.9 | <0.0001 |
| HDL, mg/dL | 46.3±10.1 | 40.0±7.4 | 43.9±9.5 | 41.2±7.8 | 44.4±9.1 | 42.2±10.2 | 42.2±8.8 | <0.0001 |
| TG, mg/dL | 129.2±67.2 | 210.5±101.6 | 150.3±75.9 | 184.2±87.3 | 145.9±68.1 | 176.3±99.1 | 173.7±102.7 | <0.0001 |
| TG/HDL | 3.7±2.8 | 3.6±1.9 | 3.6±2.5 | 3.5±2.6 | 3.9±2.6 | 3.4±2.1 | 3.7±2.7 | 0.570 |
| LDL, mg/dL | 115.5±31.2 | 108.9±28.7 | 115.4±28.8 | 115.1±33.1 | 122.4±31.2 | 116.4±33.0 | 113.5±29.1 | 0.3138 |
| HbA1c | 5.5±0.3 | 5.8±0.4 | 5.6±0.4 | 5.7±0.4 | 5.5±0.3 | 5.6±0.3 | 5.6±0.3 | <0.0001 |
| HOMA-IR | 1.3±0.5 | 3.6±1.6 | 1.4±0.5 | 3.4±1.7 | 3.7±2.8 | 1.5±0.5 | 2.0±1.2 | <0.0001 |
| HSI | 31.6±3.6 | 38.1±1.0 | 33.7±3.7 | 37.9±4.6 | 34.4±4.2 | 35.3±4.1 | 35.4±3.9 | <0.0001 |

Data are presented as number (%) or mean±SD.

^*^*P*-value using Scheffe as the post hoc analysis for comparing the groups divided by change pattern of HOMA-IR status over time.

*HOMA-IR* homeostatic model assessment for insulin resistance, *BMI* body mass index, *SBP* systolic blood pressure, *DBP* diastolic blood pressure, *GGT* gamma-glutamyl transferase, *AST* aspartate aminotransferase, *ALT* alanine aminotransferase, *HDL* high-density lipoprotein, *TG* triglyceride, *LDL* low-density lipoprotein, *HbA1c* hemoglobin A1c, *HSI* hepatic steatosis index.

**Supplementary Table S3.** Baseline characteristics in FIB-4 group

|  | All patients (n=3,047) | | | | | | | *P*-value^*^ |
| --- | --- | --- | --- | --- | --- | --- | --- | --- |
|  | **Group 1** | **Group 2** | **Group 3** | **Group 4** | **Group 5** | **Group 6** | **Group 7** |  |
| No. of patients | 803 | 445 | 324 | 253 | 33 | 554 | 635 |  |
| Age, yr | 46.1±5.4 | 59±7.4 | 48.9±7 | 55.7±7.8 | 49±8.6 | 51.4±7.6 | 52.8±7.9 | <0.0001 |
| Male sex (%) | 35.4 | 41.8 | 30.9 | 34 | 27 | 31.2 | 29.4 | 0.001 |
| BMI, kg/m^2^ | 24.9±2.9 | 23.8±3.2 | 24.9±3.1 | 24±3.2 | 23.8±3.0 | 24.8±3 | 24.4±2.9 | 0.22 |
| Waist circumference (>80 cm for women, >90 cm for men) | 81.7±8.4 | 81.7±9.1 | 81.9±8.6 | 81.6±9.5 | 79.3±9 | 82.5±8.7 | 81.9±8.8 | 0.3132 |
| SBP, mmHg | 118.9±16.2 | 126.3±17.5 | 120.2±16.6 | 126.1±19 | 117.3±17.3 | 122.7±17.8 | 123±18.8 | <0.0001 |
| DBP, mmHg | 80.3±10.8 | 82.7±10.6 | 80.2±10.7 | 82.9±12 | 78.9±10 | 81.1±10.7 | 81.6±11.8 | 0.0004 |
| Glucose, mg/dL | 82.5±8.5 | 81.2±8.2 | 81.6±7.9 | 82.1±9.2 | 79.2±8.2 | 82±7.9 | 82.1±7.90 | 0.078 |
| Creatinine, mg/dL | 0.8±0.17 | 0.8±0.15 | 0.8±0.2 | 0.8±0.2 | 0.8±0.2 | 0.8±0.2 | 0.8±0.2 | 0.2126 |
| Platelet, × mm^9^ | 304.9±61.3 | 209.1±42.4 | 284.1±52.6 | 227.1±45.4 | 214±68.6 | 272.6±52.7 | 263.4±50.7 | <0.0001 |
| Albumin, g/dL | 4.3±0.3 | 4.2±0.3 | 4.2±0.3 | 4.2±0.3 | 4.3±0.4 | 4.2±0.3 | 4.2±0.3 | <0.0001 |
| AST, U/L | 25.2±7 | 31.8±22 | 25.8±7.1 | 30.2±12.7 | 39.6±38 | 26.2±7 | 27±10.2 | <0.0001 |
| ALT, U/L | 25.4±14.7 | 26.4±30.4 | 25±14 | 23.7±15.8 | 26.4±38.7 | 20.4±16.5 | 20±18.3 | 0.1072 |
| GGT, U/L | 22.1±18.9 | 21.6±30.7 | 21.3±20.7 | 27.1±123.4 | 26.4±38.7 | 20.4±16.5 | 20.0±18.3 | 0.3617 |
| Total cholesterol, mg/dL | 190.2±33.6 | 186.6±32.6 | 190.6±34.3 | 190.7±34.6 | 183.8±27.2 | 190±33.2 | 189.1±32.9 | 0.4805 |
| HDL, mg/dL | 43.7±9.5 | 45.4±10.9 | 43.7±9.2 | 46.2±10.4 | 48.7±9.3 | 44.6±10 | 44.5±9.1 | 0.0004 |
| TG, mg/dL | 154.4±89.7 | 135.2±69.1 | 150.4±88.1 | 141.2±78.9 | 111.1±30.8 | 149.4±72.6 | 150.3±88.7 | 0.0004 |
| TG/HDL | 3.9±2.9 | 3.3±2.4 | 3.8±2.9 | 3.4±2.4 | 2.4±1.1 | 3.7±2.3 | 3.7±3 | 0.0005 |
| HbA1c | 5.5±0.3 | 5.5±0.4 | 5.5±0.3 | 5.6±0.3 | 5.6±0.4 | 5.6±0.4 | 5.6±0.4 | 0.4284 |
| HOMA-IR | 1.6±0.9 | 1.5±1.3 | 1.6±0.9 | 1.5±1.6 | 1.3±0.9 | 1.6±0.9 | 1.5±0.8 | 0.075 |
| HSI | 34.4±4.1 | 31.7±4.1 | 34.1±4.3 | 31.8±4.1 | 31.3±3.8 | 33.8±4 | 33±3.9 | <0.0001 |
| FIB-4 | 0.8±0.2 | 1.9±0.5 | 0.9±0.2 | 1.6±0.3 | 2.5±4.9 | 1±0.2 | 1.2±0.4 | <0.0001 |

Data are presented as number (%) or mean±SD.

^*^*P*-value using Scheffe as the post hoc analysis for comparing the groups divided by change pattern of FIB-4 over time.

*FIB-4* Fibrosis-4 Index for liver fibrosis, *BMI* body mass index, *SBP* systolic blood pressure, *DBP* diastolic blood pressure, *GGT* gamma-glutamyl transferase, *AST* aspartate aminotransferase, *ALT* alanine aminotransferase, *HDL* high-density lipoprotein, *TG* triglyceride, *LDL* low-density lipoprotein, *HbA1c* hemoglobin A1c, *HOMA-IR* homeostatic model assessment for insulin resistance, *HSI* hepatic steatosis index

**Supplementary Table S4.** Comparing incident DM risk between MAFLD groups

|  | **Univariate analysis** | | **Multivariate analysis (model 1)** | | **Multivariate analysis (model 2)** | |
| --- | --- | --- | --- | --- | --- | --- |
|  | **HR (95% CI)** | ***P*-value** | **HR (95% CI)** | ***P*-value** | **HR (95% CI)** | ***P*-value** |
| Group 1 | Ref |  |  |  |  |  |
| Group 2 | 5.291(4.158-6.732) | <0.0001 | 2.625 (1.727–3.989) | <0.0001 | 2.405(1.599-3.619) | <0.0001 |
| Group 3 | 1.994(1.475-2.694) | <0.0001 | 1.491 (1.087–2.047) | 0.0133 | 1.46(1.066-2.00) | 0.0185 |
| Group 4 | 2.87(2.035-4.047) | <0.0001 | 1.457 (0.95–2.234) | 0.0847 | 1.378(0.904-2.099) | 0.1356 |
| Group 5 | 2.522(1.822-3.491) | <0.0001 | 1.151 (0.762–1.738) | 0.5051 | 1.167(0.774-1.758) | 0.4605 |
| Group 6 | 1.39(0.856-2.259) | 0.1833 | 1.347 (0.816–2.224) | 0.2442 | 1.235(0.75-2.033) | 0.4066 |
| Group 7 | 3.243(2.532-4.154) | <0.0001 | 1.876 (1.371–2.566) | <0.0001 | 1.811(1.328-2.469) | 0.0002 |

Model 1 was adjusted for age, blood glucose level, ALT, AST, GGT, total cholesterol, HDL, TG, HbA1C, platelet, waist circumference, SBP, DBP, HOMA-IR, and BMI including changes in MAFLD, HOMA-IR, and BMI. Model 2 was adjusted for HbA1c, blood glucose level, HSI, TG/HDL ratio (stepwise selection), including changes in MAFLD, HOMA-IR, BMI.

*DM* diabetes mellitus, *MAFLD* metabolic associated fatty liver disease, *HR* hazard ratio, *CI* confidence interval, *ALT* alanine aminotransferase, *AST* aspartate aminotransferase, *GGT gamma-glutamyl transferase*, *HDL* high-density lipoprotein, *TG* triglyceride, *HbA1c* hemoglobin A1c, *SBP* systolic blood pressure, *DBP* diastolic blood pressure, *HOMA-IR* homeostatic model assessment for insulin resistance, *BMI* body mass index.

**Supplementary Table S5.** Comparing incident DM risk between BMI groups

|  | **Univariate analysis** | | **Multivariate analysis (model 1)** | | **Multivariate analysis (model 2)** | |
| --- | --- | --- | --- | --- | --- | --- |
|  | **HR (95% CI)** | ***P*-value** | **HR (95% CI)** | ***P*-value** | **HR (95% CI)** | ***P*-value** |
| Group 1 | Ref |  |  |  |  |  |
| Group 2 | 3.306 (2.576–4.244) | <0.0001 | 0.787 (0.492–1.260) | 0.3183 | 0.656 (0.439–0.980) | 0.0394 |
| Group 3 | 2.115 (1.319–3.389) | 0.0019 | 1.072 (0.647–1.778) | 0.7890 | 0.962 (0.586–1.578) | 0.8768 |
| Group 4 | 1.629 (0.985–2.693) | 0.0573 | 0.624 (0.346–1.128) | 0.1187 | 0.561 (0.320–0.984) | 0.0439 |
| Group 5 | 2.896 (1.933–4.340) | <0.0001 | 1.179 (0.717–1.941) | 0.5164 | 0.975 (0.613–1.551) | 0.9160 |
| Group 6 | 1.274 (0.683–2.378) | 0.4459 | 0.684 (0.353–1.328) | 0.2620 | 0.620 (0.321–1.199) | 0.1557 |
| Group 7 | 2.340 (1.583–3.458) | <0.0001 | 1.091 (0.696–1.708) | 0.7046 | 1.046 (0.679–1.611) | 0.8399 |

Model 1 was adjusted for age, blood glucose level, ALT, AST, GGT, total cholesterol, HDL, TG, HbA1C, platelet, waist circumference, SBP, DBP, BMI, HOMA-IR, and HSI including changes in HSI, HOMA-IR, and BMI. Model 2 was adjusted for HbA1c, blood glucose level, HSI, TG/HDL ratio, GGT, and DBP (stepwise selection), including changes in HSI, HOMA-IR, and BMI.

*DM* diabetes mellitus, *BMI* body mass index, *HR* hazard ratio, *CI* confidence interval, *ALT* alanine aminotransferase, *AST* aspartate aminotransferase, *GGT gamma-glutamyl transferase*, *HDL* high-density lipoprotein, *TG* triglyceride, *HbA1c* hemoglobin A1c, *SBP* systolic blood pressure, *DBP* diastolic blood pressure, *HOMA-IR* homeostatic model assessment for insulin resistance, *HSI* hepatic steatosis index.

**Supplementary Table S6.** Comparing incident DM risk between FIB-4 groups

|  | **Univariate analysis** | | **Multivariate analysis (model 1)** | | **Multivariate analysis (model 2)** | |
| --- | --- | --- | --- | --- | --- | --- |
|  | **HR (95% CI)** | ***P*-value** | **HR (95% CI)** | ***P*-value** | **HR (95% CI)** | ***P*-value** |
| Group 1 | Ref |  |  |  |  |  |
| Group 2 | 0.579(0.403-0.832) | 0.0031 | 0.787 (0.492–1.260) | 0.3183 | 0.784(0.538-1.142) | 0.2046 |
| Group 3 | 0.728(0.499-1.062) | 0.0992 | 1.072 (0.647–1.778) | 0.7890 | 0.743(0.507-1.089) | 0.1276 |
| Group 4 | 0.858(0.58-1.269) | 0.4435 | 0.624 (0.346–1.128) | 0.1187 | 0.89(0.591-1.341) | 0.5782 |
| Group 5 | 1.153(0.5082.619)- | 0.7337 | 1.179 (0.717–1.941) | 0.5164 | 1.571(0.683-1.183) | 0.2878 |
| Group 6 | 0.902(0.674-1.207) | 0.4864 | 0.684 (0.353–1.328) | 0.2620 | 0.878(0.652-1.183) | 0.3926 |
| Group 7 | 0.843(0.633-1.123) | 0.2427 | 1.091 (0.696–1.708) | 0.7046 | 0.904(0.672- 1.214) | 0.5014 |

Model 1 was adjusted for age, blood glucose level, ALT, AST, GGT, total cholesterol, HDL, TG, HbA1C, platelet, waist circumference, SBP, DBP, BMI, HOMA-IR, HSI and FIB-4 including changes in HSI, HOMA-IR, BMI and FIB-4. Model 2 was adjusted for HbA1c, blood glucose level, HSI, TG/HDL ratio, Albumin, and DBP (stepwise selection), including changes in HSI, HOMA-IR, BMI and FIB-4.

*DM* diabetes mellitus, *FIB-4* fibrosis-4 index, *HR* hazard ratio, *CI* confidence interval, *ALT* alanine aminotransferase, *AST* aspartate aminotransferase, *GGT gamma-glutamyl transferase*, *HDL* high-density lipoprotein, *TG* triglyceride, *HbA1c* hemoglobin A1c, *SBP* systolic blood pressure, *DBP* diastolic blood pressure, *BMI* body mass index, *HOMA-IR* homeostatic model assessment for insulin resistance, *HSI* hepatic steatosis index.

**
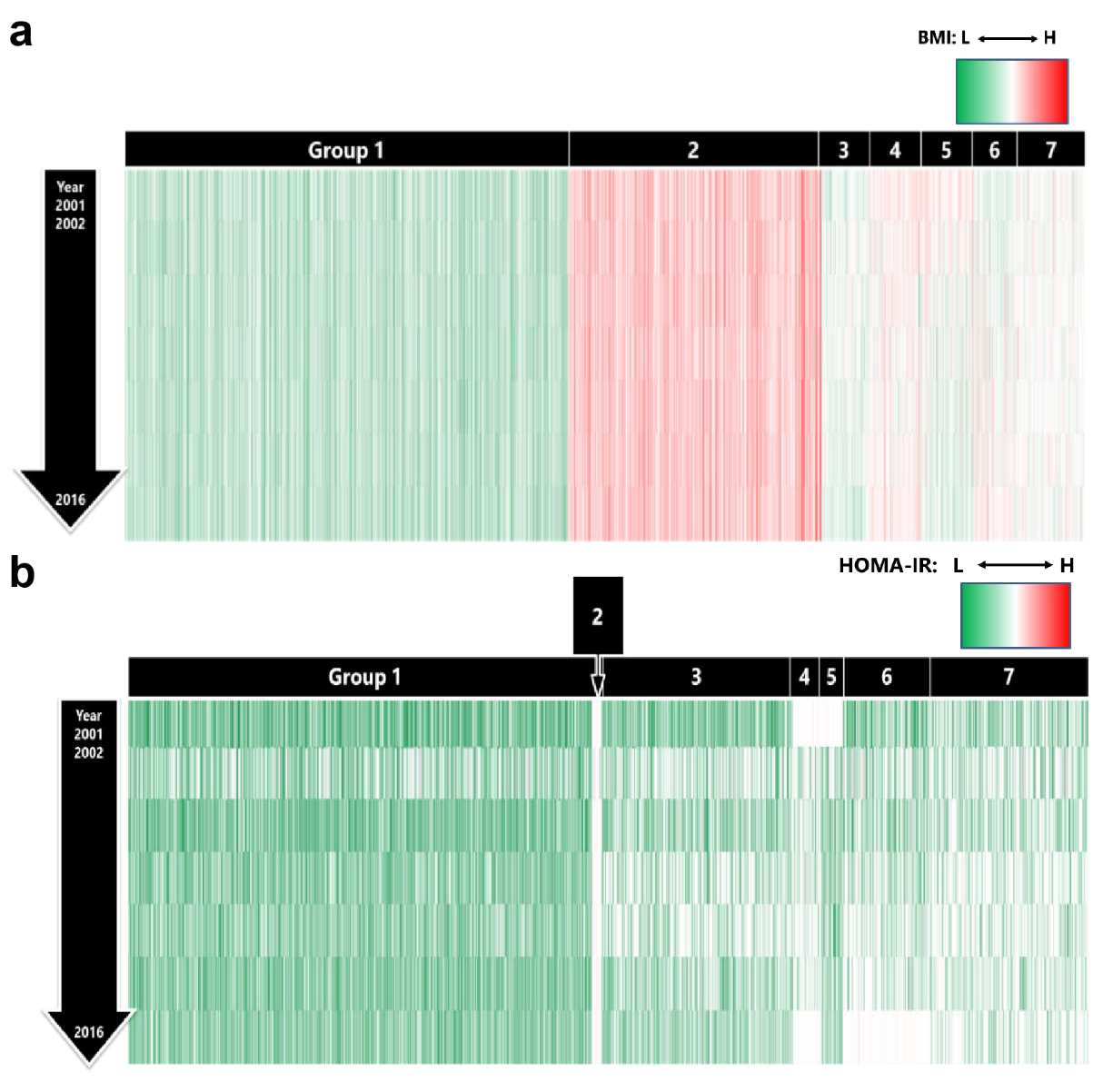
**

**Supplementary Figure S1.** Heatmaps according to BMI, HOMA-IR clustering. (**A**) Heatmaps according to BMI clustering. (**B**) Heatmaps according to HOMA-IR clustering. The y-axis represents the observed year during follow-up period and the x-axis represents the group classification. As the value for each year goes from lower to higher value, it is highlighted with green to red color in the heatmap. *BMI* body mass index, *HOMA-IR* homeostatic model assessment for insulin resistance.





**Supplementary Figure S2.** Comparison of discriminatory ability for predicting the incident diabetes mellitus (DM) between HSI change pattern over time and NAFLD development at specific time point. (**A**) Integrated area under the receiver operating characteristic curve (IAUC) plot based on HSI change pattern and NAFLD status at the first year of follow-up period in predicting incident DM, respectively. (**B**) IAUC plots based on HSI change pattern and NAFLD status at the last year of follow- up period in predicting incident DM, respectively. *HSI* hepatic steatosis index, *NAFLD* non-alcoholic fatty liver disease, *AUC* area under the receiver operating characteristic curve.
